# Minieditorial Razão Neutrófilo-Linfócito e Aterosclerose da Aorta Abdominal entre Indivíduos Assintomáticos

**DOI:** 10.36660/abc.20220182

**Published:** 2022-04-07

**Authors:** Henrique Murad

**Affiliations:** 1 Universidade Federal do Rio de Janeiro Rio de Janeiro RJ Brasil Universidade Federal do Rio de Janeiro – Cirurgia, Rio de Janeiro, RJ – Brasil

**Keywords:** Interleucina-8, Ativação de Neutrófilos, Placa Aterosclerótica/complicações, Aterosclerose, Doença da Artéria Coronariana, Inflamação, Aorta Abdominal/diagnóstico por imagem, Linfo-Histiocitose Hemofagocítica

No artigo^1^ os autores avaliam o papel da relação neutrófilo-linfócito (RNL) com a aterosclerose da aorta abdominal (AtAA). O fundamento do artigo é a premissa de que o crescimento e complicações da placa aterosclerótica são uma resposta imunologicamente mediada. Utilizam a relação neutrófilo-linfócito (RNL) como marcador inflamatório e utilizam o ultrassom da aorta abdominal para avaliar a aterosclerose subclínica, por meio dos achados de ateroma ou placa lipídica (AtAA). Os resultados entre a RNL e a síndrome coronariana aguda já foram bastante estudados, com o estado inflamatório provocando aumento dos neutrófilos e o estresse agudo secundário ao rompimento ou obstrução de placas ateroscleróticas ocasionando diminuição dos linfócitos.^2^ Machado et al. demonstraram em um grupo de 779 pacientes estudados, que o aumento da RNL em 48 a 72 horas após infarto agudo do miocárdio com elevação de ST(STEMI) estava associada com a mortalidade precoce e tardia^3^Bozkurt et al. ao estudaram 39 pacientes com Síndrome Hemofagocitica, um estado de hiperinflamação severa sistêmica, e insuficiência cardíaca com fração de ejeção preservada, observaram que a RNL era um forte preditor de mortalidade neste grupo de pacientes.^4^

Os autores analisaram 36.985 indivíduos através de ultrassom abdominal e encontraram aterosclerose da aorta abdominal em 7% deles. Este grupo de indivíduos com aterosclerose assintomática da aorta abdominal apresentava vários fatores causadores de confusão para a análise do valor da RNL como marcador de aterosclerose: sexo masculino, idade, tabagismo, diabetes, hipertensão arterial e dislipidemias.

Ao analisarem os dados de modo multivariado, ajustados para sexo, idade e fatores de risco para aterosclerose, não observaram relação entre a RNL e a AtAA. A procura de informações sobre aterosclerose em indivíduos assintomáticos através de um exame simples e de baixo custo, como a RNL é extremamente tentador. A análise multivariada realizada nesta amostra, que apresentava tantos fatores confundidores, demonstrou a ausência de relação entre a RNL e a AtAA, ou seja, entre a RNL e a aterosclerose subclinica.

A inclusão da idade como fator confundidor foi importante para definir o real papel das RNL como marcador de AtAA em individuos assintomáticos. Nos indivíduos com presença de AtAA a idade foi de 57,2+/-8,3 anos e nos indivíduos sem AtAA a idade foi de 41,2+/-9,1 anos (p<0,001). É importante incluir a idade dos pacientes ao se fazer um modelo de estudo como este

A informação mais importante desta pesquisa é verificarmos o quanto uma coleta completa de dados pode influenciar no resultado estatístico obtido. A RNL seria considerada um marcador de aterosclerose assintomática, com todas as consequências que pudessem advir desta informação, caso a idade não tivesse sido incluída.

A escolha da AtAA para a pesquisa de aterosclerose assintomática merecia alguma discussão. Li et al.,^5^ estudaram a presença de placas ateroscleróticas na aorta abdominal em um grupo de 1667 pacientes submetidos a coronariografia. Deste grupo 1268 pacientes tinham doença coronariana e 399 não a tinham. Houve uma maior prevalência de placas ateroscleróticas na aorta abdominal no grupo com doença coronariana do que no grupo sem doença coronariana (37,3% versus 17% com p<0.001). Em análise multivariada observaram que as placas abdominais foram um fator independente associada com a presença de doença coronariana. Este trabalho valida plenamente a escolha da AtAA para analisar o papel da RNL na detecção de aterosclerose assintomática.
