# Supplementary material for: Minieditorial Razão Neutrófilo-Linfócito e Aterosclerose da Aorta Abdominal entre Indivíduos Assintomáticos
Source: Arq Bras Cardiol. 2022 Apr 7;118(4):735–6. [Article in Portuguese] doi: 10.36660/abc.20220182 (PMC9007016; doi:10.36660/abc.20220182)
Supplement: Translation

# Translation: Short Editorial Neutrophil-To-Lymphocyte Ratio and Abdominal Aortic Atherosclerosis Among Asymptomatic Individuals

**Authors:** Henrique Murad

**Affiliations:** 1 Brazil Universidade Federal do Rio de Janeiro – Cirurgia, Rio de Janeiro, RJ – Brazil

**Keywords:** Interleukin-8, Neutrophil Activation, Plaque, Atherosclerotic/complications, Atherosclerosis, Coronary Artery Disease, Inflammation, Aorta Abdominal/diagnostic imaging, Lymphohistiocytosis, Hemophagocytic

In the article,^1^ the authors evaluate the role of the neutrophil-to-lymphocyte ratio (NLR) in abdominal aortic atherosclerosis (AAAt). The backbone of this paper is the knowledge that the development and complications of atherosclerotic plaques are part of an immunological response. They have used the neutrophil-to-lymphocyte ratio (NLR) as an inflammatory marker and have used abdominal aortic ultrasound to evaluate subclinical atherosclerosis through the findings of aortic atheroma or lipid plaque (AAAt). The RNL and acute coronary syndrome results were well established, with the inflammatory state leading to a rise in neutrophils and the acute stress of plaque rupture or obstruction leading to a fall in the lymphocyte count.^2^ In a group of 779 patients with ST-elevation myocardial infarction (STEMI), Machado et al. have shown a strong correlation between NLR at 48-72 hours and early and late death.^3^ Bozkurt et al. have studied 39 patients with Hemophagocytic syndrome (HPS) and heart failure with preserved ejection fraction. HPS is a state with high lethality, of severe systemic hyper inflammation with increased T lymphocytes and high levels of cytokines. In those patients, NRL was a strong predictor of mortality.^4^

The authors have studied 36.985 individuals through abdominal ultrasound and have found abdominal aortic atherosclerosis in 7% of them. In this group of abdominal aorta asymptomatic atherosclerosis, many individuals had confounding factors for NLR analysis as an atherosclerosis marker, namely age, smoking habit, diabetes, arterial hypertension, and dyslipidemia.

Through multivariate analysis adjusted for age, sex and risk factors for atherosclerosis, they have observed

no relationship between NLR and AAAt. The quest for information on asymptomatic atherosclerotic individuals through a simple and low-cost exam, like NRL, was tempting. But multivariate analysis has not shown a relationship between NRL and subclinical atherosclerosis

The inclusion of age was important to define the role of NLR as an AAAt marker in asymptomatic individuals. In AAAt positive individuals, the age was 57,2+/-8,3 years, and in the AAAt negative individuals, the age was 41.2 +/- 9.1 years (p<0.001). It is very important to include age in a study model like this one.

The most important information of this research is the need for complete data collection to have a correct statistical analysis. If age were not included in the research, NLR would be considered a marker for asymptomatic atherosclerosis, with all the consequences from this information.

The choice of AAAt in this study would deserve some discussion. Li et al.^5^ have studied abdominal aorta atherosclerotic plaques in a group of 1667 patients submitted to coronagraphy. Of those, 1268 had coronary artery disease, and 399 had not. There was more prevalence of atherosclerotic plaques in the coronary artery disease group than in the no coronary artery disease group (37,3% vs. 17%, p<0,001). In multivariate analysis, abdominal aortic plaques were an independent factor associated with coronary artery disease. This paper brings validation to the choice of AAAt to analyze the role of NLR in asymptomatic atherosclerosis
